# A new fossil dolphin *Dilophodelphis fordycei* provides insight into the evolution of supraorbital crests in Platanistoidea (Mammalia, Cetacea)

**DOI:** 10.1098/rsos.170022

**Published:** 2017-05-31

**Authors:** Alexandra T. Boersma, Matthew R. McCurry, Nicholas D. Pyenson

**Affiliations:** 1 Department of Paleobiology, National Museum of Natural History, Smithsonian Institution, PO Box 37012, Washington, DC 20013, USA; 2 College of Extended Education, California State UniversityMonterey Bay, Seaside, CA 93955, USA; 3 Monash Biomedicine Discovery Institute and Centre for Human Anatomy Education, Department of Anatomy and Developmental Biology, Monash UniversityMelbourne, Victoria 3800, Australia; 4 School of Biological Sciences, Monash University, Melbourne, Victoria 3800, Australia; 5 Geosciences, Museums Victoria, Melbourne, Victoria 3053, Australia; 6 Department of Mammalogy, Burke Museum of Natural History and Culture, Seattle, WA 98195, USA; 7 Department of Paleontology, Burke Museum of Natural History and Culture, Seattle, WA 98195, USA

**Keywords:** Cetacean, Platanistoidea, river dolphins, Miocene, pneumatization, computed tomography

## Abstract

Many odontocete groups have developed enlarged facial crests, although these crests differ in topography, composition and function. The most elaborate crests occur in the South Asian river dolphin (*Platanista gangetica*), in which they rise dorsally as delicate, pneumatized wings anterior of the facial bones. Their position wrapping around the melon suggests their involvement in sound propagation for echolocation. To better understand the origin of crests in this lineage, we examined facial crests among fossil and living Platanistoidea, including a new taxon, *Dilophodelphis fordycei*, nov. gen. and sp., described herein, from the Early Miocene Astoria Formation of Oregon, USA. We measured the physical extent and thickness of platanistoid crests, categorized their relative position and used computed tomography scans to examine their internal morphology and relative bone density. Integrating these traits in a phylogenetic context, we determined that the onset of crest elaboration or enlargement and the evolution of crest pneumatization among the platanistoids were separate events, with crest enlargement beginning in the Oligocene. However, we find no evidence for pneumatization until possibly the Early Miocene, although certainly by the Middle Miocene. Such an evolutionary context, including data from the fossil record, should inform modelling efforts that seek to understand the diversity of sound generation morphology in Odontoceti.

## 1. Introduction

Cetacean skulls are dramatically different in construction from their closest mammalian relatives, largely reflecting adaptation to demands imposed by life in water versus ancestry on land. Many cetacean skull elements are posteriorly ‘telescoped’, a term referring to the elongation of rostral elements and posterior movement of caudal elements, resulting in the overlap of facial bones that were once adjacent in ancestral configurations more typical of land mammals [1]. In odontocetes, telescoping has layered the maxillae over the frontals in the antorbital/supraorbital region of the face. Many odontocete taxa (including *Physeter macrocephalus* Linnaeus, 1758 [2], *Hyperoodon ampullatus* Forster, 1770 [3] and *Inia geoffrensis* Blainville, 1817 [4]) also have elevated facial crests of varying degrees in the antorbital/supraorbital region, composed of the frontals and maxillae. Hypotheses on the function of these crests vary from taxon to taxon, including a range from structural support (e.g. *Physeter*) to a head-butting apparatus (e.g. *Hyperoodon*, Ziphiidae) [5]. Equally, facial crests may serve as attachment surfaces for the origin of facial and rostral muscles [6,7].

The most elaborate facial crests occur in the South Asian river dolphin (*Platanista gangetica*) (Lebeck, 1801 [8]), where they rise dorsally above the level of the nuchal crest in the supraorbital region as extremely thin wings, and extend anteriorly to the level of the antorbital notches. *Platanista*'s crests are unique among cetaceans both because of their relative size and because they are completely pneumatized. The pterygoid air sac system, which is usually limited to the ventral side of the skull, invades dorsally and lines the entire medial surface of the supraorbital crests [9,10]. The crests also wrap around the melon, a fatty organ involved in sound generation. Both the position and pneumatization of the crests suggest their involvement in the generation or propagation of sound in the head, though this hypothesis is difficult to test both experimentally, and even more so logistically considering the endangered status of this species [11,12].

To better understand the evolutionary origin of the crests and pneumatization in this lineage, we examined the supraorbital crests in a group phylogenetically allied with *Platanista*. Termed Platanistoidea, this group of cetaceans consists of taxa whose stratigraphic range spans from the mid-Oligocene to the present, including almost exclusively marine forms except for *Platanista* [13]. We measured the relative size of the crests, analysed their cranial element composition and characterized their shape. In addition, we used computed tomography (CT) scanning to examine the internal morphology and relative bone density of the crests.

Results show that almost all members of the group Platanistidae (*sensu* [13]), including a new taxon *Dilophodelphis* Boersma, McCurry & Pyenson, 2017, have elevated crests that show varying amount of pneumatization. More basal branching platanistoids, and the basal-most platanistid *Araeodelphis* Kellogg, 1957 [14], show small supraorbital crests, and with no patent pneumatization. *Dilophodelphis* has crests that are highly elevated, but also show no clear signs of pneumatization. The remaining platanistid species, i.e. *Pomatodelphis inaequalis* Allen, 1921 [15], *Zarhachis flagellator* Cope, 1868 [16] and *Platanista gangetica*, all show elevated supraorbital crests with some pneumatization. *Both* crest elevation and pneumatization evolved only once among platanistoids (in the lineage leading to the clade Squalodelphinidae + Platanistidae), although at different times. Also, *Platanista* is unique in the size and shape of its crests, which probably concentrate sound reflections during the signal generation phase of underwater echolocation.

## 2. Material and methods

### 2.1. Data collection

To examine and compare internal supraorbital crest morphology, we selected candidate specimens from putative platanistoid groups (see Boersma & Pyenson [13] for a discussion), which were then scanned using Nikon Metrology's combined 225/450 kV microfocus X-ray and CT walk-in vault system at Chesapeake Testing in Belcamp, MD, USA. Using the vault CT scanner system, we collected slices at 0.63 mm, resulting in three-dimensional reconstruction increments of 0.30 mm. We used this instrumentation to CT scan the holotype of *Dilophodelphis fordycei* (USNM 214911), as well as specimens of *Squalodon calvertensis* Kellogg, 1923 [17] (Squalodontidae, USNM 328343), Squalodelphinidae gen. and sp*.* indet. (Squalodelphinidae, USNM 475496) and *Arktocara yakataga* Boersma and Pyenson, 2016 [13] (Allodelphinidae, USNM 214830). All platanistid taxa with enlarged supraorbital crests were also scanned for finer scale comparisons, including *Platanista gangetica*, *Pomatodelphis inaequalis* and *Zarhachis flagellator.* The latter two taxa were scanned using a Siemens SOMATOM Emotion 6 CT scanner located at the Smithsonian Institution Bio-Imaging Research Center in the Department of Anthropology at the National Museum of Natural History, in Washington, DC. One *Platanista* specimen (USNM 23456) that we examined had the left supraorbital crest separated from the skull at a clean break, which was carefully fitted to the skull for scanning. The DICOM files produced from all the scans were examined in ImageJ [18] and processed in Mimics (Materialize NV, Leuven, Belgium) to create three-dimensional (3D) surface models. The model of *Dilophodelphis* is available for viewing and download on the Smithsonian X 3D website (http://3d.si.edu). The original DICOM files for the holotype USNM 214911 are archived on Zenodo (http://zenodo.org) at the following (doi:10.5281/zenodo.232492).

### 2.2. Phylogenetics

We tested the phylogenetic placement of *Dilophodelphis* using a modified version of a platanistoid matrix published by Godfrey *et al*. [19], which was modified from Lambert *et al*.'s [20] original matrix as a base (table 2). Godfrey *et al*.'s [19] version consisted of 41 morphological characters and 21 operational taxonomic units (OTUs) including the fossil platanistoid genera *Huaridelphis* Lambert, Bianucci and Urbina, 2014 [20], *Notocetus* Moreno, 1892 [21], *Phocageneus*, *Squalodelphis* Dal Piaz, 1917 [22], *Medocinia* Muizon, 1988 [23], *Zarhachis*, *Pomatodelphis*, *Zarhinocetus* [24], *Ninjadelphis ujiharai* Kimura and Barnes, 2016 [25], *Allodelphis* Wilson, 1935 [26], *Araeodelphis* and the extant *Platanista*. Also included were the contested platanistoids, *Waipatia* Fordyce, 1994 [27], and *Squalodon*. We added three genera: *Goedertius* Kimura and Barnes, 2016 [25], *Arktocara* and *Otekaikea* Tanaka and Fordyce, 2014 [28], and removed four taxa that were not relevant for our analysis, resulting in 20 OTUs. We altered character 48 (pneumatic maxillary crest overhanging medially), making it a multi-state character to account for the varying degrees of pneumatization as seen in *Zarhachis* and *Pomatodelphis* versus *Platanista* (see discussion for further comment on differing pneumatization). We also removed one character (character 4 in Godfrey *et al*. [19]) that we found subjective and problematic, and added 27 morphological characters, resulting in a total of 67 characters used in the analysis (table 3).

### 2.3. Functional analysis

We used CT scans of extant and fossil platanistids to examine the relative bone density of three regions of the skull: the ventral section of the rostrum which is composed of maxillae, the supraorbital crests and the zygomatic processes of the squamosal. We avoided collecting data on bone density from the dorsal portion of the premaxillae, which can be hyper-ossified [54,55]. We used the ‘Draw profile line’ function in Mimics V 18 (Materialize NV, Leuven, Belgium) to collect lateral profiles in Hounsfield Units (a scale of radiodensity). This method provided a way to estimate relative density within each CT scan but it did not allow for density measures to be directly compared between specimens. This limitation is acceptable because our goal was to evaluate the difference in bone density between the supraorbital crests and the rest of the skull in each individual specimen.

### 2.4. Nomenclature acts

This published work and the nomenclatural acts it contains have been registered in ZooBank, the online registration system of the International Code of Zoological Nomenclature. The ZooBank LSIDs (Life Science Identifiers) can be viewed by appending the LSID to the prefix ‘http://zoobank.org/’. The LSID for this publication is: urn:lsid:zoobank.org:pub:E8D93F41-EBFC-46AF-B2E0-08C2FE484B86. The electronic edition of this work was published in a journal with an ISSN, and has been archived and is available from the following digital repositories: PubMed Central and LOCKSS.

### 2.5. Specimens observed

*Allodelphis pratti* (YPM 13408); *Allodelphis* sp. (USNM 266608, 256609, 256610); *Araeodelphis natator* (USNM 526604); *Arktocara yakataga* (USNM 214830); *Goedertius oregonensis* (LACM 123887); *Goedertius* sp. (USNM 335406, 335765, 13673, 314421); *Notocetus* sp. (USNM 206286); *Phocageneus venustus* Leidy, 1869 [56] (USNM 21039); *Phocageneus* sp. (USNM 182939, 362125); *Platanista gangetica* (USNM 23456); *Pomatodelphis bobengi* Case, 1934 [57] (USNM 299775); *Pomatodelphis* sp. (USNM 360054); Squalodelphinidae gen. and sp. indet. (USNM 475496); *Squalodon calvertensis* (USNM 10949, 529246); cast of *Waipatia maerewhenua* (USNM 508061); *Zarhachis flagellator* (USNM 299945, 10911, 13768); *Zarhachis* sp. (USNM 214759, 24868); cast of *Zarhinocetus errabundus* (USNM 526600); *Zarhinocetus errabundus* (USNM 11573, 25425).

## 3. Results

### 3.1. Systematic palaeontology

Cetacea Brisson, 1762 [58]

Odontoceti Flower, 1867 [59] *sensu* Fordyce and Muizon, 2001 [11]

Platanistoidea *sensu* Boersma and Pyenson 2016 [13]

Platanistidae Gray, 1846 [60] *sensu* Boersma and Pyenson 2016 [13].

*Dilophodelphis*, gen. nov. LSID: urn:lsid:zoobank.org:act:212FAC29-AF67-4E93-B04A-1E96A9817270

*Type and only included species*. *Dilophodelphis fordycei*, sp. nov.

*Etymology*. From the Greek words *di* (double), *lophos* (crest) and *delphis* (dolphin), referring to the enlarged supraorbital crests on the dorsal surface of the skull, resembling twin mountain crests. This construction also evokes the dinosaur *Dilophosaurus wetherilli* Welles 1954 [61], a double-crested theropod recovered from Early Jurassic sequences of the Kayenta Formation in Arizona, USA.

*Age*. Same as that of the species.

*Diagnosis*. Same as that of the species.

**Figure 1.**
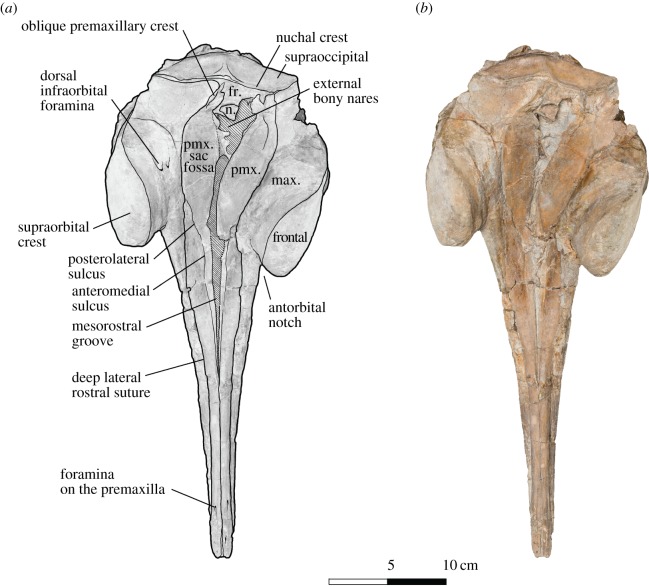
Skull of *Dilophodelphis* (USNM 214911) in dorsal view. (*a*) Illustrated skull with low opacity mask, interpretive line art and labels for skull elements. Dotted lines indicate uncertainty of sutures, and dashed lines highlight fossae. Hatched pattern indicates areas where sediment is obscuring the fossil. (*b*) Photograph of skull in dorsal view, photography by James Di Loreto, Smithsonian Institution. fr., frontals; max., maxilla; n., nasal; pmx., premaxilla; pmx. sac fossa, premaxillary sac fossa.

**Figure 2.**
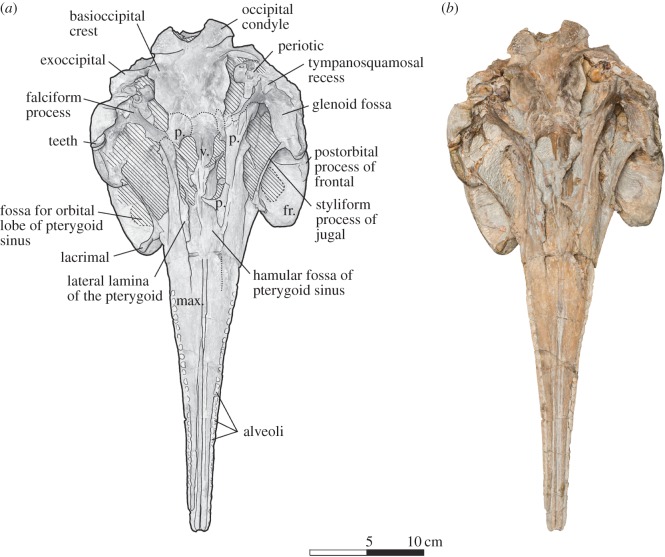
Skull of *Dilophodelphis* (USNM 214911) in ventral view. (*a*) Illustrated skull with low opacity mask, interpretive line art and labels for skull elements. Dotted lines indicate uncertainty of sutures, and dashed lines highlight fossae. Hatched pattern indicates areas where sediment is obscuring the fossil. (*b*) Photograph of skull in ventral view, photography by James Di Loreto, Smithsonian Institution. fr., frontal; max., maxilla; p., pterygoid; v., vomer.

**Figure 3.**
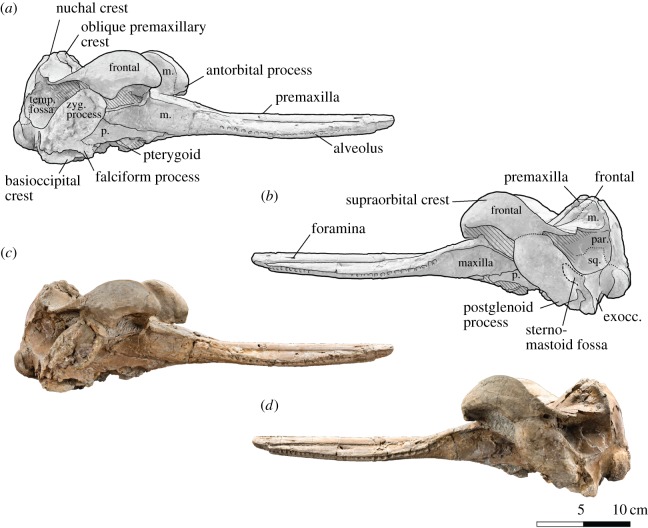
Skull of *Dilophodelphis* (USNM 214911) in right and left lateral views. (*a*) Illustrated skull in right lateral view and (*b*) left lateral view with low opacity mask, interpretive line art and labels for skull elements. Dotted lines indicate uncertainty of sutures, and dashed lines highlight fossae. Hatched pattern indicates areas where sediment is obscuring the fossil. (*c*) Photograph of skull in right lateral view and (*d*) left lateral view, photography by James Di Loreto, Smithsonian Institution. exocc., exoccipital; m., maxilla; par., parietal; p., pterygoid; sq., squamosal; temp. fossa, temporal fossa; zyg. process, zygomatic process.

**Figure 4.**
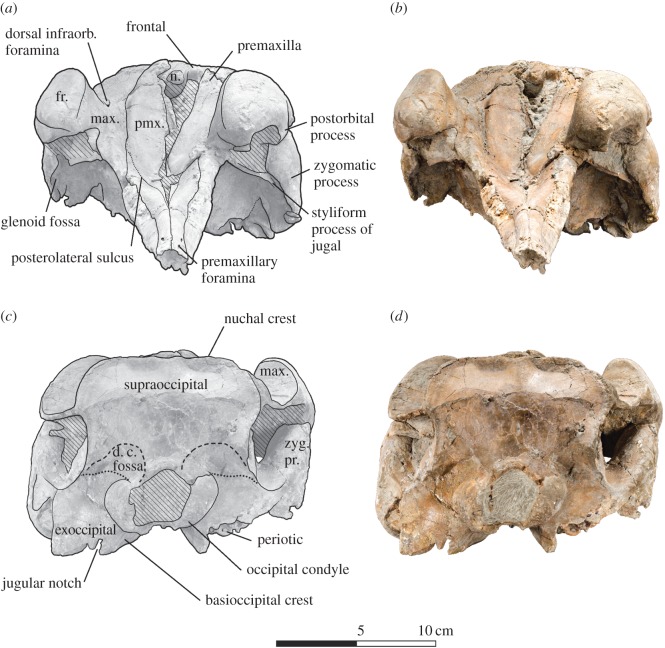
Skull of *Dilophodelphis* (USNM 214911) in anterior and posterior views. (*a*) Illustrated skull in anterior and (*c*) posterior view with low opacity mask, interpretive line art and labels for skull elements. Dotted lines indicate uncertainty of sutures, and dashed lines highlight fossae. Hatched pattern indicates areas where sediment is obscuring the fossil. (*b*) Photograph of skull in anterior view and (*b*) posterior view, photography by James Di Loreto, Smithsonian Institution. fr., frontal; d. c. fossa, dorsal condyloid fossa; max., maxilla; pmx., premaxilla; zyg. pr., zygomatic process.

**Figure 5.**
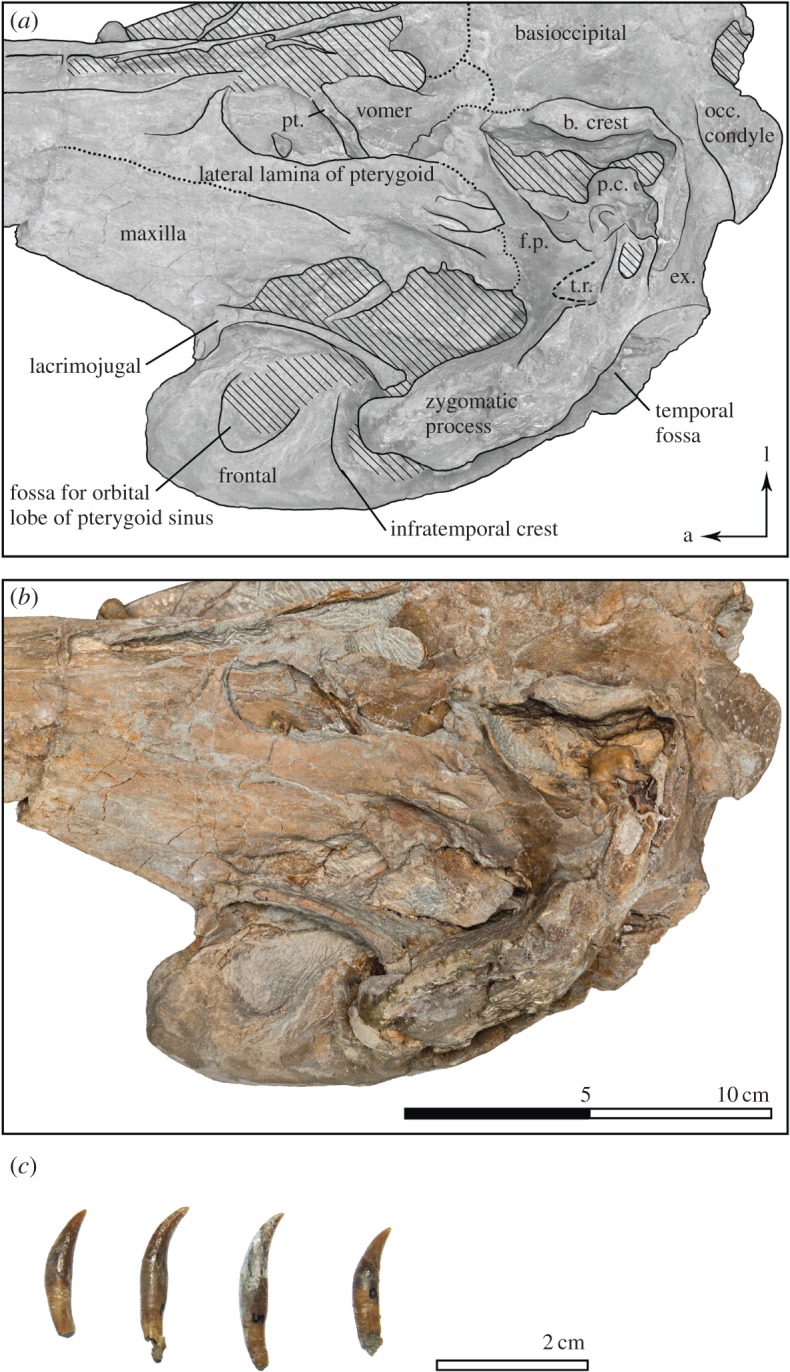
Detail of *Dilophodelphis* (USNM 214911) in ventral view and dentition. (*a*) Illustrated detail of the right half skull in ventral view with low opacity mask, interpretive line art, and labels for skull elements. Dotted lines indicate uncertainty of sutures, and dashed lines highlight fossae. Hatched pattern indicates areas where sediment is obscuring the fossil. (*b*) Photograph of right half of skull in ventral view, photography by James Di Loreto, Smithsonian Institution. (*c*) Photographs of isolated teeth belonging to USNM 214911, found in the surrounding matrix. b. crest, basioccipital crest; ex., exoccipital; f.p., falciform process; occ. condyle, occipital condyle; p.c., pars cochlearis; pt., pterygoid; t.r., tympanosquamosal recess. Arrows indicate anatomical direction: a, anterior; l, left lateral.

**Figure 6.**
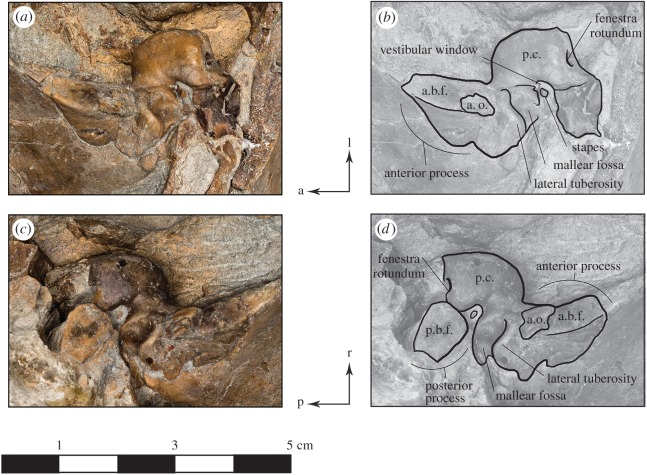
Periotics of *Dilophodelphis* (USNM 214911) in ventrolateral views. (*a*) Photograph of right periotic and (*c*) photograph of left periotic, photography by James Di Loreto, Smithsonian Institution. (*b*) Illustrated right and (*d*) left periotics with low opacity mask, interpretive line art and labels for skull elements. a.o., accessory ossicle; a.b.f., anterior bullar facet; p.b.f., posterior bullar facet; p.c., pars cochlearis. Arrows indicate anatomical direction: a, anterior; l, left lateral; p, posterior; r, right lateral.

*Dilophodelphis fordycei*, sp. nov. (figures 1–6, tables 1–3)

**Table 1.** Measurements of USNM 214911 (in centimetres).

dimension	measurement (cm)
total preserved length of skull from furthest anterior point to furthest posterior point	44
cranial length from antorbital notches to occipital condyle	17.2
rostral length from anterior tip to antorbital notch	28.4
distance between upper margin of foramen magnum and nuchal crest	6.8
height of foramen magnum	3.5
height of supraorbital crest	5.4
height of temporal fossa	5.5
height of rostrum at base	5.2
length of temporal fossa	8.7
orbit length	5.4
nasal length	1.3
length of vertex (nuchal crest to anterior transverse margin of nasal)	2.8
width of rostrum between antorbital notches	7.9
width of premaxillae at rostrum base	2.2
maximum width of premaxillae on cranium	7.7
width of external bony nares (dorsal)	2.8
width of external bony nares (ventral)	3.8
postorbital width of skull	18
bizygomatic width of skull	18.8
width of combined nasals	2.5
width of nuchal crest	12
width of foramen magnum	3.4
width of occipital condyles	7.3
width of exoccipitals	15.2
width of anterior tip of rostrum	1.6

**Table 2.** Matrix constructed in Mesquite for Platanistoidea, .pdf format 0, primitive state; 1, 2, 3, derived states; 0 and 1, a variable between 0 and 1; 1 and 2, a variable between 1 and 2; ? missing character or taxon not coded for this character.

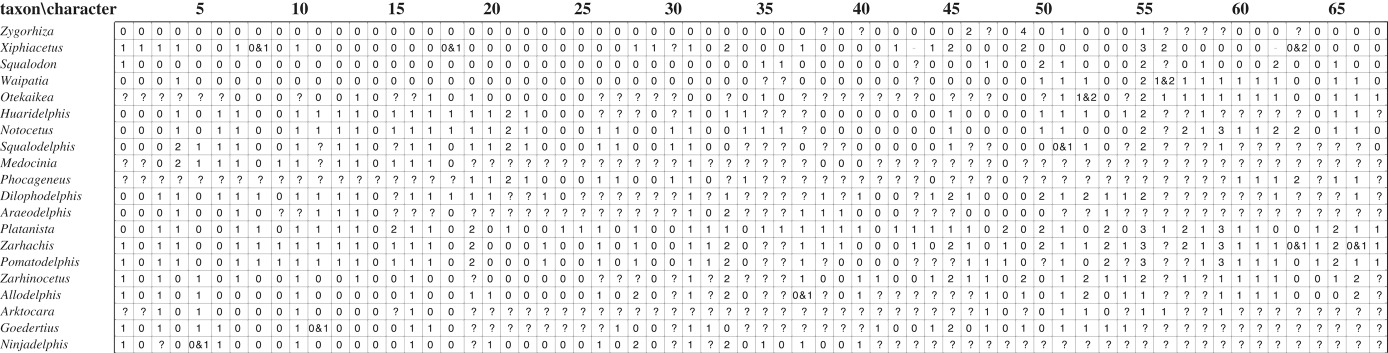

**Table 3.** Character state descriptions following Godfrey *et al*. [19] and Tanaka & Fordyce [28].

List of characters used in the phylogenetic analysis. Characters are polarized with respect to *Zygorhiza* as the outgroup. Modified from Godfrey *et al*. [19].	
1.	Rostrum elongation (Bianucci *et al*. [29], modified): short, ratio between rostrum length and condylobasal length < 0.70 (0); elongated, ratio > 0.70 (1)
2.	Apex of the rostrum constituted by the only premaxillae on more than 10% of its total length and lacking alveoli [30]: absent (0); present (1)
3.	Lateral rostral suture between premaxilla and maxilla deeply grooved [27]: no (0); yes (1)
4.	Widening of the premaxillae at the rostrum base: narrow premaxillae, ratio between the width of the rostrum and the transverse width of the premaxillae at the antorbital notch < 0.60 (0); wide premaxillae, ratio between 0.60 and 0.75 (1); extremely wide premaxillae nearly reaching the lateral margin of the rostrum, ratio > 0.75 (2)
5.	Dorsal opening of the mesorostral groove anterior to the rostrum base [31] (modified): narrower than the premaxilla (0); wider than the premaxilla (1)
6.	Deep, ‘V’-shaped, left antorbital notch, related to an anteriorly pointed antorbital process: no (0); yes (1)
7.	Elevated antorbital region, distinctly higher than the dorsal margin of the rostrum base in lateral view: no (0); yes (1)
8.	Distinct dorsal crest in the antorbital–supraorbital region: no (0); yes (1)
9.	Thickening of the antorbital process of the frontal, quantified as a ratio between the height of the antorbital process of the frontal, measured in lateral view perpendicular to the maxillary-frontal suture above the orbit, and the vertical distance from the lower margin of the occipital condyles to the vertex of the skull; absent, ratio < 0.25 (0); present, ratio > 0.30 (1)
10.	Widening of the cranium: cranium roughly as long as wide or longer than wide with ratio between cranium length (longitudinal, from occipital condyles to level of antorbital notches) and postorbital width > 0.90 (0); cranium distinctly shorter than wide with ratio < 0.90 (1)
11.	Posterior infraorbital foramen(ina) along the vertex more medial than the lateral-most margin of the premaxilla in the cranium: no (0); yes (1)
12.	Deep fossa in the frontal on orbit roof, at the level of the frontal groove: no (0); yes (1)
13.	Vertex distinctly shifted to the left compared with the sagittal plane of the skull: no (0); yes (1)
14.	Transverse premaxillary crest on the vertex [30]: absent (0); present (1)
15.	Ventral exposure of the palatine [32] (modified): palatine widely exposed anterior to the pterygoid (0); palatine only exposed laterally to the lateral lamina of the pterygoid (1); palatine completely covered by the pterygoid (2)
16.	Hamular fossa of the pterygoid sinus [33]: short, not reaching anteriorly the level of the antorbital notch (0); long, extending anteriorly on the palatal surface of the rostrum (1)
17.	Thickening of the zygomatic process of the squamosal: absent, ratio between the maximum distance from the anteroventral margin of the zygomatic process to the posterodorsal margin, in lateral view and the vertical distance from the lower margin of the occipital condyles to the vertex of the skull < 0.35 (0); present, ratio > 0.35 (1)
18.	Circle-shaped dorsal outline of the zygomatic process of the squamosal in lateral view: no (0); yes (1)
19.	Articular rim on the lateral surface of the periotic [32] (modified): absent (0); present (1); present and hook-like (2)
20.	Pars cochlearis of the periotic square-shaped in ventral view [32]: no (0); yes (1)
21.	Aperture of the cochlear aqueduct of the periotic [32], modified; [7]: small (0); very small (1); large and thin-edged (2)
22.	Aperture of the cochlear aqueduct of the periotic [32] (modified): faces mediodorsally (0); faces dorsally (1)
23.	Transverse thickening of the anterior process of the periotic [32]: no (0); yes (1)
24.	Internal auditory meatus of the periotic oval, with the dorsal opening for the facial canal lateral to the spiral cribriform tract [7]: no (0); yes (1)
25.	Separate ossicle at the apex of the anterior process of the periotic [7]: no (0); yes (1)
26.	Elongated anterior spine on the tympanic bulla, associated to a marked anterolateral convexity [32]: no (0); yes (1)
27.	Ventral groove of the tympanic affecting the whole length of the bone, including the anterior spine [32]: no (0); yes (1)
28.	Extent of the inner and outer posterior prominences of the tympanic: both prominences with approximately the same posterior extent (0); outer posterior prominence posteriorly longer than the inner posterior prominence (1); outer posterior prominence posteriorly shorter than the inner posterior prominence (2)
29.	Dorsal margin of the involucrum of the tympanic cut by a median indentation, in medial view [30]: absent (0); present (1)
30.	Apical extension of the manubrium of the malleus [32]: no (0); yes (1)
31.	Loss of double-rooted posterior teeth [32]: no (0); yes (1)
32.	Retention of accessory denticles on posterior teeth [32] (modified): yes (0); no (1)
33.	Tooth count per upper or lower row: <25 (0); >25 and <33 (1); >33 (2)
34.	Strong development of the dorsal transverse process of the atlas and extreme reduction of its ventral process [32]: no (0); yes (1)
35.	Great reduction of coracoid process of the scapula [32–34]: no (0); yes (1)
36.	Great reduction or loss of supraspinatus fossa, with acromion located on anterior edge of scapula [32,34,35]: no (0); yes (1)
37.	Deep lateral groove on mandible [1]: no (0); yes (1)
38.	Medial margin of the antorbital notch made of a thin plate: no, robust lateral margin of the rostrum at base (0); yes (1)
39.	Dorsal surface of vertex: flat (0); markedly transversely and longitudinally convex (1)
40.	Vertex strongly transversely pinched: absent (0); present, maxillae converging markedly posterior to bony nares (1)
41.	Lateral margin of rostrum anterior to maxillary flange: concave (0); straight (1); convex (2); absent (3) ([36] #9, [37,38] #7; modified from Bianucci [39] #3)
42.	Suture between maxilla and premaxilla on rostrum: unfused except distal tip of rostrum (0); fused partly or along most of rostrum (1). ([36] #15, [37,38] #14; modified from Fordyce [27] #36; [40] #1418; [31] #10; [30] #2; [41,42] #10)
43.	Upper anterior ‘teeth’: about same size as upper posterior teeth (0); greatly enlarged (1); clearly smaller than upper posterior teeth or absent (2). ([36] #22, modified from Murakami *et al*. [37,38] #22)
44.	Cheek teeth entocingulum: present (0); absent (1). ([36] #24, [31] #32; [41,42] #32; [37,38] #24)
45.	Greatest diameter of largest functional tooth as percentage of greatest width of maxillae at the level of the postorbital processes: large, >5% (0); medium, 5–3% (1); small, <3% (2). ([36] #25, [37,38] #25; modified from Aguirre-Fernández *et al*. [43] #15)
46.	Antorbital process of maxilla in dorsal view: triangular (0); robust and globose or rectilinear (1); absent (2). ([36] #34, [39] #4; [37,38] #34)
47.	Posterolateral sulcus: deep (0); shallow or absent (1); presence of additional posterolateral sulcus (longitudinal striation) (2). ([36] #57, [37,38] #55; modified from de Muizon [23,44]; [45] #6; [31] #72; [41,42] #72)
48.	Pneumatic maxillary crest overhanging medially: absent (0); present as a deep air pocket in-tucking from the ventral side, as seen in *Pomatodelphis* and *Zarhachis* (1); present, air spaces lining the interior surface of the supraorbital crests, as in *Platanista* (2). ([36] #65; [46]; [6] #26; [47] #58; [27] #66; [48] #14; [40] #1421; [37,38] #63)
49.	Maxillary crest on supraorbital process of maxilla: longitudinal ridges absent except at lateral edge of antorbital process (0); the presence of longitudinal ridge except at lateral edge of antorbital process (1); longitudinal ridge present and joined with maxillary flange (2); the presence of transversely compressed high crest, except at lateral edge of antorbital process (3); absent (4). ([36] #66, [37,38] #64; modified from de Muizon [32,44]; [49]; [40] #1420; [31] #79; [41,42] #79; derived from Miller [1])
50.	Premaxillary cleft: absent (0); present, posterior part of ascending processes of premaxillae bearing a distinct cleft, originating at posterior edge of premaxillae and continuing anteriorly, dividing each premaxilla into two (1); present, with shallow cleft (2). ([36] #85, [31] #109; [41,42] #109; [37,38] #84)
51.	Zygomatic process of squamosal: directed anterolaterally (0); directed anteriorly (1). ([36] #109, [50]; [31] #142; [41,42] #142; [37,38] #108)
52.	Emargination of posterior edge of zygomatic process by neck muscle fossa, skull in lateral view: absent, posterior edge forming nearly right angle with dorsal edge of zygomatic process of squamosal (0); shallow emargination (1); deep emargination (2). ([36] #111, [31] #144; [41,42] #144; [37,38] #110)
53.	Ventral edge of zygomatic process of squamosal in lateral view: concave (0); almost straight (1); convex (2). ([36] #113, [31]; #150; [41,42] #150; [37,38] #112)
54.	Lateral lamina of palatine: absent (0); present (1). ([36] #122, [23,34,44]; [48] #16; [40] #1443; [37,38] #121)
55.	Tympanosquamosal recess: absent, with anterior transverse ridge present (0); anterior transverse ridge absent and middle sinus inferred to be present without a large tympanosquamosal recess (1); present and enlarged, forming triangular fossa medial and anteromedial to postglenoid process (2); very large, forming large fossa bordering entire medial edge of glenoid fossa (3). ([36] #144, [31] #178; [41,42] #178; [37,38] #143; modified from Lambert [30] #35; derived from Fraser and Purves [9] and Fordyce [51])
56.	Position of more-distal part of alisphenoid–squamosal suture, with skull in ventral view: anterior to external opening of foramen oval or a homologous groove (0); courses along groove for mandibular branch of trigeminal nerve, or just posterior to it (1); just medial to anterior edge of floor of squamosal fossa, foramen ovale and/or groove situated entirely on alisphenoid (2). ([36] #147, [31] #180; [41,42] #180; [37,38] #146)
57.	Suprameatal pit of squamosal: absent (0); present but shallow, situated dorsolateral to spiny process of squamosal (1); forming deep dorsolateral excavation into squamosal (2). ([36] #149, [31] #185; [41,42] #185; [37,38] #149)
58.	Foramen spinosum: absent (0); present, located in anteromedial corner of anterior part of periotic fossa near or on squamosal–parietal suture (1). ([36] #150, [35]; [31] #186; [41,42] #186; [37,38] #150)
59.	Posterior portion of periotic fossa of squamosal: fossa absent (0); fossa present but shallow (1); highly compressed fossa forming narrow slit or small blind foramen (2); posteromedial portion contains large deep fossa (3). ([36] #151, [31] #187; [41,42] #187; [37,38] #149 and #151)
60.	Lateral groove or depression affecting profile of periotic as viewed dorsally: no obvious vertical groove dorsal to hiatus epitympanicus (0); groove present with overall profile of periotic becoming slightly to markedly sigmoidal in dorsal view (1). ([36] #166, [27] #35; [37,38] #166)
61.	Anteroposterior ridge on dorsal side: undeveloped (0); developed on anterior process and body of periotic, associated with development of depression adjacent to groove for tensor tympani (1). ([36] #167, [27] #55; [37,38] #167)
62.	Parabullary sulcus: absent (0); strongly curved, C-shape (1); weakly curved (2); strongly curved, V-shape (3). ([36] #169, modified from Fordyce [27] #56 Anteroexternal sulcus)
63.	Aperture for cochlear aqueduct: smaller than aperture for vestibular aqueduct (0); approximately same size as aperture for vestibular aqueduct (1); much larger than aperture for vestibular aqueduct, with narrow posterior edge (2). ([36] #180, [31] #227; [41,42] #227; [37,38] #181; modified from de Muizon [32]; [27]; [30] #52)
64.	Excavation of tegmen tympani at base of anterior process: absent (0); present, with fossa on dorsolateral side of tegmen tympani (1). ([36] #181, [31] #231; [41,42] #231; [37,38] #182)
65.	Articular rim: absent (0); present but small, forming ridge anterolateral to articulation surface of posterior process of periotic and separated from it by sulcus (1); present, sigmoidal and laterally elongated with hook-like process (2). ([36] #186, [31] #239; [41,42] #239; modified from Murakami *et al*. [37,38] #187; modified from de Muizon [32]; [52]; [40] #1494; [27] #33; [30] #55)
66.	Ventral surface of posterior process of periotic, along a straight path perpendicular to its long axis: flat (0); concave (1); convex (2). ([36] #191, [37,38] #191; modified from Geisler and Sanders [31] #242; [41,42] #242)
67.	Posterior edge of medial prominence of involucrum: approximately in line with posterior edge of lateral prominence (0); distinctly anterior to posterior edge of lateral prominence (1). ([36] #209, [32]; [31] #269; [41,42] #269; [37,38] #209; derived from Kasuya [53])

LSID: urn:lsid:zoobank.org:act:49967F02-61C0-45E9-8165-D303CFFBE78B

*Holotype*. USNM 214911 consists of an almost complete skull retaining both periotics, but missing both tympanic bullae, the very distal tip of the rostrum (not more than approx. 1 cm), both mandibles, fragments of the basicranium, and all dentition except for several isolated teeth that were originally prepared out of the matrix by Douglas R. Emlong. While none of the teeth remain in the rostrum, based on archival notes on the preparation and condition of this specimen at USNM, we are confident that these isolated teeth are indeed associated with this taxon because of their matching alveolar size and placement close to the skull, including the size and morphological correspondence of three isolated teeth that still are in contact with zygomatic process of the squamosal. In November 1970, Emlong collected a second specimen, both mandibles of an unknown cetacean, found in a drift concretion directly in front of the spot in the cliff where USNM 214911 was found. However, based on the mismatched size of the mandibles relative to USNM 214911, we can definitively exclude them from belonging to the holotype of *Dilophodelphis*.

*Type locality*, *formation and age*: The holotype was recovered by Emlong in December 1967. Emlong's notes indicate the locality of Nye Beach, OR, USA, about 0.4 km south of the Jump-Off Joe site and 0.4 km north of what was then the Hotel Gilmore. Jump-Off Joe was a sea stack formation of Middle Miocene concretionary sandstone, located at about 44.6454° N, 124.0626° W. Hotel Gilmore, originally the New Cliff House and now the Sylvia Beach Hotel, is a U.S. National Register Historic Place sitting on a bluff above Nye Beach, at 44.6383° N, 124.0621° W [62]. Based on Emlong's notes, the type locality of USNM 214911 should be halfway between these two locations, at approximately 44.6417° N, 124.0626° W. Emlong reported that the skull was found in the Astoria Formation, a Miocene marine sequence exposed along the seacliffs north and south of Yaquina Head in Newport, Oregon, though Emlong did not document more specific information about the stratigraphic position of the holotype. South of Yaquina Head, the lower part of the formation is exposed, and is approximately 130 m thick [63]. Benthic foraminifera sampled from the Astoria Formation in the Newport area were mostly Saucesian in age [63] and molluscs sampled from the formation further constrain it to 20.7–15.1 Ma. Molluscs specifically from the Jump-Off Joe site were Early Miocene in age, between 20 and 19 Ma. Thus, the age of the type USNM 214911 is probably approximately between 20 and 19 Ma or Early Burdigalian (Late early Miocene).

#### 3.1.1. Differential diagnosis

*Dilophodelphis* is a small- to medium-sized odontocete (approx. 2.27 m in total length), which belongs to Platanistoidea (*sensu* [13]) based on two synapomorphies: emargination of the posterior edge of the zygomatic process by the sternomastoid muscle fossa with the skull in lateral view (character 52[2]) and ventral surface of the posterior process of the periotic, concave along a straight path perpendicular to its long axis (character 66[1]). Character 52 shows state reversals among the platanistoids. The remaining four platanistoid synapomorphies recovered in our phylogenetic analysis are either not preserved or obscured by matrix in the holotype of *Dilophodelphis*.

*Dilophodelphis* differs from all known Waipatiidae, Squalodelphinidae and Allodelphinidae in having a distinct dorsal crest in the antorbital–supraorbital region (character 8[1]). *Dilophodelphis* differs from waipatiids and squalodelphinids in having a deeply grooved lateral rostral suture between the premaxilla and the maxilla (character 3[1]); in having a small tooth diameter as a percentage of postorbital skull width (character 45[2]); in having the robust and globose/rectilinear antorbital processes of the maxilla in dorsal view (character 46[1]) and in having the ventral edge of the zygomatic process almost straight in lateral view (character 53[1]). *Dilophodelphis* further differs from waipatiids and allodelphinids by having an elevated antorbital region distinctly higher than the dorsal margin of the rostrum base in lateral view (character 7[1]); having a deep fossa in the frontal on the orbit roof at the level of the frontal groove (character 12[1]) and having a circle-shaped dorsal outline of the zygomatic process of the squamosal in lateral view (character 18[1]). *Dilophodelphis* differs from waipatiids and other platanistids in having a square-shaped pars cochlearis of the periotic (character 20[1]).

*Dilophodelphis* also differs from the Waipatiidae in having a deep, ‘V’-shaped antorbital notch in relation to an anteriorly pointed antorbital process (character 6[1]); having a cranium distinctly shorter than wide with a ratio <0.90 (character 10[1]); wide hamular fossa of the pterygoid sinus, extending anteriorly onto the palatal surface of the rostrum (character 16[1]); transverse thickening of the anterior process of the periotic (character 23[1]) and in having a lateral lamina of the palatine (character 54[1]).

*Dilophodelphis* differs from the Allodelphinidae in lacking an elongated rostrum (character 1[0]), having wide premaxillae at the rostrum base (character 4[1]), having the dorsal opening of the mesorostral groove anterior to the rostrum base narrower than the premaxilla (character 5[0]), having a concave lateral margin of the rostrum anterior to the maxillary flange (character 41[0]), having a deep posterolateral sulcus (character 47[0]), having a shallow premaxillary cleft (character 50[2]) and having the ventral surface of the posterior process of the periotic concave along a straight path perpendicular to its long axis (character 66[1]).

*Dilophodelphis* belongs to the Platanistidae based on the following: medial margin of the antorbital notch made of a thin plate (character 38[1]), and dorsal surface of the vertex markedly transversely and longitudinally convex (character 39[1]). *Dilophodelphis* also shares the following characteristics with all the platanistids, excluding *Araeodelphis*: lateral rostral suture between premaxilla and maxilla deeply grooved (character 3[1]), distinct dorsal crest in the antorbital–supraorbital region (character 8[1]), antorbital process of maxilla robust and globose or rectilinear in dorsal view (character 46[1]), and the presence of a shallow premaxillary cleft (character 50[2]).

*Dilophodelphis* differs from all other known platanistids in having an enlarged tympanosquamosal recess forming a large triangular fossa medial and anteromedial to the postglenoid process (character 55[2]). *Dilophodelphis* demonstrates one clear apomorphy: a deep emargination of the posterior edge of the zygomatic process by the sternomastoid fossa, in lateral view (character 52[2]). Further features that distinguish *Dilophodelphis* from other platanistids are discussed in the ‘Morphological comparisons’ section of the discussion. Finally, *Dilophodelphis* demonstrates the following possibly plesiomorphic character states (shared with at least some squalodelphinids): deep, ‘V’-shaped antorbital notch in relation to an anteriorly pointed antorbital process (character 6[1]); semicircular dorsal outline of the zygomatic process of the squamosal in lateral view (character 18[1]); pars cochlearis of the periotic square-shaped in ventral view (character 20[1]).

#### 3.1.2. Etymology

The species epithet honours Prof. R. Ewan Fordyce, FRSNZ, native New Zealander and prominent vertebrate palaeontologist. The epithet recognizes his extensive and long-lasting contributions to the field of marine mammal palaeontology, including his commitment to mentoring future scientists, especially in shaping the career paths of the authors herein. The epithet also honours his long-standing interest in the fossil marine mammal record of Oregon, which has yielded pivotal specimens for over a century, including *Simocetus rayi* Fordyce 2002 [51], which he described.

### 3.2. Abbreviated description

The following outlines the key features of the holotype, with terminology following Mead & Fordyce [54]; for all measurements of the skull (table 1). The skull is nearly complete, measuring 44 cm in maximum sagittal length, and missing only the very distal tip of the rostrum (not more than approx. 1 cm), the lower jaws, the tympanic bullae and fragments of the basicranium (figures 1–6). Several teeth were found in the surrounding matrix adjacent to the skull, but none were retained in the rostrum itself (figure 5). Also found separated from the skull were the left nasal and an isolated fragment of the left paroccipital process. The entire skull is asymmetrically skewed in a variety of different ways. While the asymmetry in the facial region is likely original, based on comparison with other platanistoids, the asymmetrical skew of the rostrum from the sagittal plane is probably attributable to diagenesis.

#### 3.2.1. Dorsal view

The rostrum is practically complete, missing 1 cm from the distal end at most and moderate in length, measuring approximately 28.3 cm from the anterior tip to the right antorbital notch (figures 1–3). Both rostral premaxillae bear a small foramen, approximately 5 cm from the preserved rostral tip, with the foramina on the right premaxilla slightly further anterior than the left. The left premaxilla may possibly exhibit a second, small foramen, less than 2 cm from the anterior tip of the rostrum, but it is less distinct than the other two. Anteriorly, the left and right premaxillae remain in contact along the midline and roof over the mesorostral groove for approximately half the rostrum length, until approximately 16.5 cm posterior of the tip, where the two premaxillae separate laterally to slightly expose a narrow slit of the mesorostral groove. From the mesorostral groove, at this level, and heading posterolaterally, the anteromedial sulcus runs down the premaxilla, dividing the bone into a narrow strip medially and a wider section laterally. The anteromedial sulcus transitions into the posterolateral sulcus approximately 1 cm before the level of the right antorbital notch. The premaxillary foramen would be expected at this meeting point between the two sulci; however, the preservation of the skull has left no clear evidence of a foramen on either side. Also at the level of the anterior opening of the mesorostral groove, the premaxillae switch from being dorsally elevated above the maxillae (anterior) to being ventral of the maxillae, as the maxillae rise dorsally to form a basin on the rostral base (figure 3). This basin continues posteriorly onto the facial surface of the skull, formed ventroposteriorly by the wide, flat and depressed premaxillae and laterally by the elevated supraorbital crests composed of maxillae and frontals. Mead & Fordyce ([54]: Diagram 2) provided a thorough discussion on the different morphologies of the platanistoid supraorbital crests, and other elevated features in the antorbital region of other cetaceans. In *Dilophodelphis*, the elevated supraorbital crests extend anteroposteriorly from the antorbital process to a level between the postorbital process and the nuchal crest (figure 3). They are robust and rounded dorsally, with the maxillae forming the medial face and the frontal forming the lateral face. The premaxillae continue to widen transversely from the rostral base to approximately the level of the postorbital processes, at which point they maintain a regular width until the level of the external nares (figure 1). Heading posteriorly from the external nares towards the vertex, the premaxillae contract transversely and rise dorsally above the maxillae once again. Both premaxillae are asymmetrically skewed to the left, with the right one possessing a distinct oblique crest on the lateral margin of the posterolateral surface. The external bony nares are obscured by matrix, but are small and appear to be oriented slightly posterior of vertical. The left nasal is disassociated from the skull, but it probably was in contact medially with the right nasal, which is roughly square in dorsal view, and rests on the vertex in a fossa of the frontal. The left, disassociated nasal is thicker medially, narrowing in dorsoventral depth laterally. The right nasal is separated from the premaxilla by a small exposure of frontal, possibly due to breakage of the lateral edge of the nasal. The frontals are exposed on the vertex as an anteroposteriorly narrow rectangle between the nasals and the nuchal crest. The vertex is dorsally elevated to the level of the nuchal crest, and the temporal fossae are obscured in dorsal view by the laterally extended temporal crest formed by maxilla and frontal.

#### 3.2.2. Lateral view

While the anterior tip of the rostrum is composed solely of premaxilla in the preserved specimen, it is possible that the maxilla did originally contribute to the anterior rostral extremity (a typical platanistid character state, as described by Godfrey *et al*. [19]. In lateral view, the alveolar groove of the premaxilla bows slightly ventrally as it heads posteriorly, before distinctly curving dorsally as it nears the base of the rostrum. The supraorbital crest is at the same dorsal height as the nuchal crest, and is convex dorsally along its dorsal margin (figure 3). The posteroventral margin of the crest, posterior of the postorbital process, is almost in contact with the anterodorsal edge of the zygomatic process of the squamosal, separated only by a thin layer of matrix. In lateral view, the zygomatic process is very robust, roughly rectangular and pointed anterodorsally, with a nearly straight ventral edge. The postglenoid process is rounded off. The medial wall of the temporal fossa is laterally convex, and its circumference is roughly shaped like a spear tip, with a narrowly pointed anterior margin and a shallowly pointed posterior margin, in lateral view. Both lacrimals are present, and the ventral edge of the antorbital process and antorbital notch appears to be composed of the lacrimojugal. However, this area is somewhat obscured by the matrix supporting the thin styliform process of the jugal, which extends from the antorbital notch to the lateral edge of the glenoid fossa of the squamosal (though this positioning is likely to be due to diagenetic displacement, as the jugal generally ends anterodorsolaterally to the glenoid fossa in crown odontocetes).

#### 3.2.3. Posterior view

In posterior view, the supraoccipital shield forms a broadly pentagonal shape, with a transverse constriction dorsal to the level of the occipital condyles exposing the temporal fossa (figure 4*c*). Though the shield is generally posteriorly convex, there are deep fossae immediately posterior to the nuchal crest, as well as shallower fossae medial to the transverse constriction. The exoccipitals also bear deep dorsal condyloid fossae. The squamosal is widely exposed lateral of the exoccipital in posterior view. The occipital condyles are posteriorly pedestaled, and their surfaces are pitted, suggested that the specimen may be physically immature [64]. The jugular notch is deep and narrow, associated with a robust paroccipital process representing the ventral-most part of the skull.

#### 3.2.4. Ventral view

In ventral view, the rostral maxilla bears two deep grooves medial to the alveolar groove on either side, reaching from the rostral tip to approximately 17.5 cm posterior (figure 2). A third groove extends down the midline of the rostrum where the maxillae come into contact, widening approximately at the same level 17.5 cm posterior of the rostral tip to expose a long, thin section of the vomer. From the base of the rostrum to the anterior basicranium, the vomer rises as a ventrally pointed keel down the midline of the skull, flanked on either side anterior to the external bony nares by the medial lamina of the pterygoids, and further anterior by the palatines, which are broken to expose the hamular fossa of the pterygoid sinus. This fossa is shallow and extremely elongated anteriorly, reaching at least 10 cm in anteroposterior length. The pterygoid borders this fossa laterally, forming a lateral lamina that extends from the palate posteriorly to contact the falciform process of the squamosal. The limits of the palatines could not be detected, either because of closed sutures or because they are hidden under the pterygoid. The orbital roof, formed by the frontal, is deeply excavated by a round fossa for the pterygoid sinus, posteriorly bordered by a thick infratemporal crest. The glenoid fossa on the zygomatic process is relatively shallow, and there is a deep, triangular tympanosquamosal recess. The basioccipital crests are thick and wavy (potentially because of diagenetic deformation), oriented at a low angle from the sagittal plane and ventrolaterally tilted to overhang the posterior lacerate foramen and foramen ovale in ventral view.

#### 3.2.5. Dentition

Though the anterior alveoli are somewhat damaged and obscured by matrix, there were probably approximately 35 teeth per tooth row in the rostrum. No teeth remain in the alveoli, though a number were found in the surrounding matrix and two teeth are still stuck to the left glenoid fossa by matrix. These teeth are homodont, with the longest 2.1 cm in length, and are extremely delicate, with a pointed cusp on a crown approximately 0.6 cm in height that has extremely fine (if any) longitudinal striae.

#### 3.2.6. Periotics

Both periotics remain *in situ*, and are so closely appressed to the squamosal to prevent removal. However, we provide a description of the details that can be observed, either from the right or left periotic, which are 33 and 32 mm long, respectively. The anterior process is both inflated and elongate relative to the pars cochlearis, with a relatively flat anterior bullar facet, as observed on the right periotic. Laterally, the profile of the anterior process is pinched anteriorly, as far as can be determined with its anatomical position *in situ*, hemmed in by the margin of the squamosal. The lateral tuberosity of the periotic forms a transverse crest that is sigmoidal in shape, with an approximate anteroposterior thickness of about 2 mm. The lateral tuberosity forms the anterior border of the mallear fossa, which is somewhat ovate. The pars cochlearis is generally rectangular in ventral profile, with the fenestra rotundum (or round window) at a right angle from the fenestra ovalis (or vestibular window). Both stapes are associated with openings for the vestibular windows, although they are clearly broken and we did not remove them from the surrounding sediment for this study. Although not entirely visible in their current orientation, we can confirm that both periotics have a large, dorsally oriented aperture for the cochlear aqueduct. Lastly, the length of the posterior process of the periotic does not extend beyond the lateral margin of the anterior process.

The posterior process exhibits a slightly pinched quadrangular outline, with a smooth posterior bullar facet.

### 3.3. Body size estimate

Total body length (TL) was estimated using the body size estimation formula created by Pyenson & Sponberg [64] for stem Platanistoidea (*sensu* [64]) based on the bizygomatic width (BIZYG) of the skull:

Log(L)=0.92×(log⁡(BIZYG)−1.51)+2.49.

The BIZYG of *Dilophodelphis* is 18.8 cm, producing a reconstructed body length of 2.27 m.

### 3.4. Phylogenetic analysis

**Figure 7.**
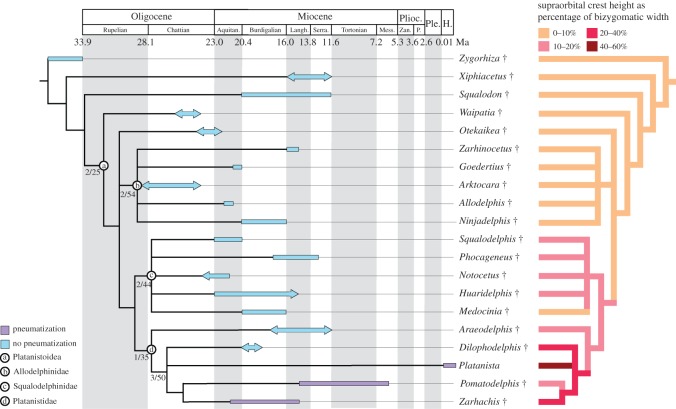
Calibrated and supraorbital crest size phylogenetic trees. On the left is a time-calibrated phylogenetic tree of the Platanistoidea, showing the node-based clade of Platanistoidea and the node-based families Allodelphinidae, Squalodelphinidae and Platanistidae, along with their support values (Bremer decay index/bootstrap value). Coloured bars correspond to the stratigraphic ranges of each taxon, with arrows indicated lower confidence in stratigraphic boundaries. Stratigraphic range data was derived from published accounts for each taxon, including global ranges. Geologic time scale based on Cohen *et al*. [65]. The absence or presence of pneumatization is indicated by bar colour (blue for absence, purple for presence). Labelled circles denote node-based clades. Abbreviations: Aquitan., Aquitanian; H., Holocene; Langh., Langhian; Mess., Messinian; P., Piacenzian; Ple., Pleistocene; Plioc., Pliocene; Serra., Serravallian; Zan., Zanclean. The right tree shows the evolution of supraorbital crest size in platanistoids, calculated as a percentage of BIZYG and binned by 4 colour values. See electronic supplementary material, table S1 for dataset of crest measurements used.

The phylogenetic analysis, conducted in TNT, resulted in 65 most parsimonious trees, with a best score of 150, consistency index of 0.57 and retention index of 0.71. The strict consensus tree resolved two possibilities for a node-based Platanistoidea: the first including *Waipatia*, *Otekaikea*, *Ninjadelphis*, *Goedertius*, *Arktocara*, *Allodelphis*, *Zarhinocetus*, *Araeodelphis*, *Platanista*, *Dilophodelphis*, *Pomatodelphis*, *Zarhachis*, *Phocageneus*, *Medocinia*, *Squalodelphis*, *Notocetus* and *Huaridelphi*s, and the second also including *Squalodon* (figure 7). We elected to use the first possibility, excluding *Squalodon*, as displayed in our figures, as it yielded slightly higher support (decay index 2, bootstrap 25). However, the inclusion or exclusion of *Squalodon* in Platanistoidea should be tested with a larger sample of platanistoids, putative platanistoids and basal odontocetes. In our results, we resolved previously supported clades of Allodelphinidae, Squalodelphinidae and Platanistidae (see [13]), with Squalodelphinidae and Platanistidae as sister groups. *Dilophodelphis* is placed in a polytomy with *Platanista* and the clade of *Pomatodelphis *+ *Zarhachis*, which may correspond to the clade Pomatodelphininae, as defined by Barnes [66] and Bianucci *et al*. [67]. We recovered *Araeodelphis* as the most basal lineage in this clade of Platanistidae. Our analysis yields moderate support for the monophyly of Allodelphinidae and Squalodelphinidae (decay index 2, bootstrap > 55), with lower support for the clades of Platanistoidea (decay index 2, bootstrap 25) and Platanistidae (decay index 1, bootstrap 35).

### 3.5. Crest comparison

#### 3.5.1. Cranial elements

**Figure 8.**
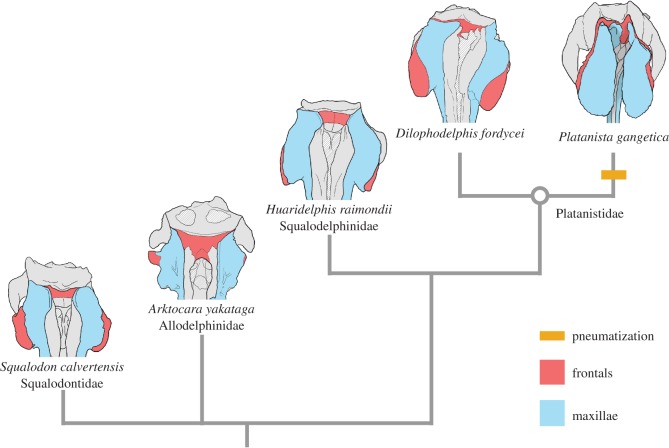
Configuration of maxillae and frontals in the supraorbital region. Simplified phylogeny of the platanistoid families (with Squalodontidae as an outgroup), highlighting the configuration of maxilla and frontal bones on the facial region. The maxillae are highlighted in blue, and the frontals are highlighted in red. The yellow marker denotes the appearance of pneumatization in the supraorbital crests of the platanistids.

Our first step to characterizing platanistoid facial crests involved determining what cranial elements contributed to the overall composition of the crests in different taxa (figure 8). In our survey, we determined that the crests are composed of maxillae, frontals, or both. In basal branching platanistoids (i.e. waipatiids, squalodontids and allodelphinids), which exhibited little to no enlargement of the supraorbital crests, the supraorbital region is composed of relatively equal parts of frontals and overlying maxillae. This ratio also holds true for squalodelphinids, which show some enlargement of the crests, but with the maxillae still completely overlying the frontals. The platanistid *Araeodelphis* shows a crest condition more similar to the squalodelphinids, with modest crest enlargement and maxillae overlying the frontals. However, in platanistid taxa such as *Pomatodelphis*, *Zarhachis* and *Dilophodelphis*, with enlarged crests, they are composed of frontals on the lateral faces and maxillae on the medial faces. These two cranial elements meet along the dorsal ridge of each crest, at an indistinct suture. *Platanista* is the only platanistoid where the crests are composed exclusively of maxillae. In the case of *Platanista*, the maxillae have extended dorsally and separated into two thin laminae, separated by trabeculae and curving medially to wrap around the melon [9].

#### 3.5.2. Asymmetry

Another changing morphological characteristic to note among platanistoids is the varying asymmetry in the skull (figure 8). Facial asymmetry, focused around the narial passages, is typical in odontocete skulls, with multiple proposed functions including a role in sound production, and an adaptation to protect the respiratory tract while swallowing large prey items whole [68–70]. In platanistoids, this skull asymmetry extends to the facial crests, and in varying degrees among different putative subclades. In squalodontids and allodelphinids, the skull and supraorbital area are only slightly asymmetrical, but in squalodelphinids and *Araeodelphis* both the relative size and position of the bilateral skull elements are strongly skewed sinistrally extending to the antorbital notches and the premaxillae at the rostrum base. This cranial asymmetry is even more extreme among the remaining platanistids, especially in *Dilophodelphis* and *Platanista*, where the left supraorbital crest extends much further anteriorly than the right, and many of the right vertex elements are completely skewed to the left, beyond a midline plane defined by the underlying cerebral hemispheres.

#### 3.5.3. Crest internal surface shape

We examined the shape of the supraorbital crests of *Dilophodelphis* relative to other platanistids with elevated crests (i.e. excluding *Araeodelphis*, whose crest condition is more similar to that of squalodelphinids). Assuming that elaborate crests are involved in sound production and propagation during sound generation, we propose that the internal surface would contribute to reflecting sound waves generated by the phonic lips, which are located dorsomedial of the crests in *Platanista*. We observed that the crests and the corresponding air sacs of *Platanista* are unique among platanistoids (and all fossil and living odontocetes) in having inward crest surfaces that are parabolic, opening anteroventrally (electronic supplementary material, figure S1). By contrast, the medial surfaces of the crests in *Pomatodelphis* and *Zarhachis* are almost vertical, and in *Dilophodelphis* they are parabolic again, but opening dorsally. Basic acoustic principles suggest a differential function to the reflective properties of concave versus convex surfaces [71]. Convex surfaces (e.g. the medial crest surface in *Dilophodelphis*) distribute reflected sound much more widely than concave surfaces (e.g. *Platanista*'s crest surface), especially at high frequencies. Concave surfaces tend to concentrate reflected sound. As a result, if both supraorbital crests participate in sound production/propagation for platanistoids, the range of surface concavity/convexity and orientation would produce very different results for sound propagation in the soft tissues of the forehead. Lastly, we note that the ventromedial surface of the crests of *Platanista* is highly rugose, a characteristic that would also influence the reflective properties of the crests. More complex surfaces provide a relatively larger surface for sound to interact with, and thus function better as sound absorbers and sound diffusers than as sound reflectors [72]. However, if the interface between the air sac system and the soft tissue acts to reflect sound, then the surface of this bone may not be important in determining the reflective properties of this system. Lastly, in this regard, it is difficult to make definitive statements about the acoustic properties without knowing the point source of sound, which differs in different lineages of odontocetes and is unknown for *Platanista*.

#### 3.5.4. Crest density

We quantified cross-sectional density of the facial crests relative to the rostrum and the zygomatic process of the squamosal. We used the latter feature as a consistent comparative baseline. In all three platanistoids for which this comparison could be made (*Dilophodelphis fordycei*, *Platanista gangetica* and *Pomatodelphis inaequalis*), we determined that the crests and the maxillary section of the rostrum shared similar high densities, while the zygomatic process was considerably less dense. The crests of *Dilophodelphis* are slightly more relatively dense compared with the other two species (figure 10).

#### 3.5.5. Relative size

We determined the relative supraorbital crest size by measuring the greatest height of the crest, and calculated it as a percentage of BIZYG (see electronic supplementary material, table S1 for supraorbital crest measurements). Height was measured from the dorsal-most point on the supraorbital crest to the ventral-most point on the orbit roof, often the postorbital process. Postorbital process length does not vary substantially enough among platanistoids to effect our relative size measurements. BIZYG has been upheld as the most reliable proxy for condylobasal length, and was used because many of the fossil skulls used are fragmentary [64]. *Platanista* has by far the largest supraorbital crests, measuring 58% of BIZYG. *Dilophodelphis* has the next largest crests, at only 28% of BIZYG. Pilleri & Gihr [73] provided a similar quantification of crests, though without providing nearly as specific details delimiting landmarks for the morphometric measurements of the crests. Also, without corresponding skull measurements (indeed, some of their reported vouchers may be lost), their data cannot be directly compared with ours.

#### 3.5.6. Pneumatization

**Figure 9.**
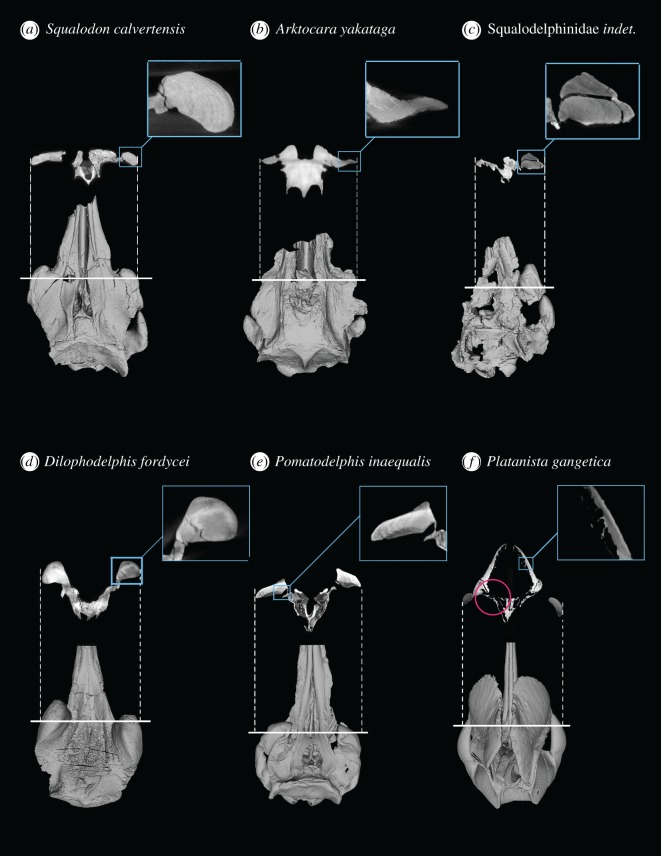
The presence and absence of pneumatization from CT data. CT scan cross-sections of platanistoids from each of the families (with *Squalodon* as an outgroup). White solid line across 3D model shows level of the skull at which the CT scan was taken (across the supraorbital crest). Blue boxes show enlarged images of the supraorbital crest. Red circle around the cross section of *Platanista* highlights the connection between the pterygoid sinus system and the pneumatic spaces lining the medial supraorbital crests.

We used CT data to determine whether there was any evidence of pneumatization in the supraorbital crests of platanistoids other than *Platanista.* The three basal-most platanistoids scanned (*Squalodon*, *Arktocara* and a squalodelphinid) showed no pneumatization of the crests (figure 9). Cross-sectional CT scans at the level just anterior to the antorbital notch in these taxa showed that the supraorbital area is composed of solid bone (figure 9). The modest supraorbital eminences in *Araeodelphis* appear to be devoid of pneumatization, based on the observations made by Godfrey *et al*. [19]. In the referred specimen of *Araeodelphis* (USNM 10478), as in squalodelphinids, the pterygoid sinus excavates a deep fossa on the ventral surface of the frontal reaching to the outer margin of the orbit roof, but it does not penetrate and open onto the dorsal side of the skull. *Dilophodelphis* also has a deep fossa for the pterygoid sinus on the orbital roof of the frontal. Cross-sectional scans of *Dilophodelphis*' crests were patched with dark shadows, probably representing areas of lower density bone that could be precursors to pneumatic spaces. However, these cannot be considered pneumatic spaces, and for the purposes of this paper we define *Dilophodelphis* as being devoid of pneumatization (see Discussion for further comments on pneumatization). All the remaining members of the clade Platanistidae (i.e. *Pomatodelphis*, *Zarhachis* and *Platanista*) demonstrate some evidence of pneumatization, though in different ways. As previously stated, the crests of *Platanista* are composed of two maxillary laminae, joined by trabeculae and invaded by air spaces of the pterygoid air sac system [9]. The CT scans of *Pomatodelphis* showed that the crest has an air pocket in-tucking from the ventral side, corresponding to a much deeper fossa for the orbital lobe of the pterygoid sinus when compared with squalodelphinids, *Araeodelphis* and *Dilophodelphis* [20,74]. The slightly crushed appearance of the medial surface of facial crests in nearly every specimen of *Pomatodelphis* suggests that, in life, the medial crest surfaces were probably closed by a thin layer of bone. However, it is possible that these air pockets communicated openly with soft anatomy of the facial region, in a manner perhaps homologous with the sinus connections of *Platanista* (Figure 9). *Zarhachis* shows the same crest condition as *Pomatodelphis*.

## 4. Discussion

### 4.1. Supraorbital crests and pneumatization

Facial crests are common traits in the supraorbital region of odontocetes ([54], table 1), yet their disparate composition and placement challenges interpretations of their homology and function. Among platanistoids, supraorbital crests are most evident among two nominal groups: squalodelphinids and platanistids. Allodelphinids and waipatiids show supraorbital crests generally on par with most delphinoids ([54], fig. 1), whereas the thickness and topography of supraorbital crests in squalodelphinids and platanistids are distinctive features of the facial region. Squalodelphinids show no evidence of pneumatization of the supraorbital crests, and this also holds true for *Araeodelphis* and *Dilophodelphis*. Though phylogenetically a platanistid, *Araeodelphis* has crests only slightly more enlarged than squalodelphinids. It is also worth mentioning that *Prepomatodelphis* Barnes, 2002 [75] possibly lacked supraorbital crests, although damage to the specimen in the supraorbital region makes a clear determination of this impossible, and we did not have access to the specimen for this study. *Platanista* possesses the largest supraorbital crests of any platanistoid, based on pure linear morphometrics, with those of *Dilophodelphis* ranking second in size, and *Zarhachis* close behind. Compositionally, the crests of *Platanista* are composed solely of the maxillae, which is a unique condition among platanistoids—all others have crests composed of both the maxillae and frontals. The supraorbital crests of *Dilophodelphis*, *Pomatodelphis* and *Zarhachis* are composed of relatively equal surfaces of the frontals and maxillae, with the maxillae forming the internal surfaces and the frontals forming the exterior surfaces (figure 8). Internally, there are clear indications that all the platanistids, excluding *Araeodelphis* and *Dilophodelphis*, show some degree of pneumatization in the supraorbital crests. Any future studies to test the role of *Platanista*'s supraorbital crests in sound production should take into consideration all the crest characteristics outlined above, and how they have changed phylogenetically and through geologic time.

Considering the differences in putative pneumatization among *Pomatodelphis*, *Zarhachis* and *Platanista*, the question arises of how to define pneumatization. In *Pomatodelphis* and *Zarhachis*, the fossa for the pterygoid sinus is excavated so deeply that there are three clearly defined bony walls of the cavity. In *Platanista*, the pneumatic air spaces are contained between the two thin, bony lamina of the maxilla. Therefore, we define pneumatic spaces simply as the extension of air spaces inside the crests, differentiating them from the pterygoid sinus fossa seen in the squalodelphinids, *Araeodelphis* and *Dilophodelphis.* The discovery of more platanistids will surely help to clarify the transitional states between *Araeodelphis/Dilophodelphis*, *Pomatodelphis/Zarhachis* and *Platanista*. With new data, more states will need to be added to phylogenetic analyses to account for the variation of pneumatized states among platanistids. For now, we have defined three: lack of pneumatization in *Araeodelphis* and *Dilophodelphis*; moderate pneumatization in *Pomatodelphis* and *Zarhachis*; and extensive pneumatization in *Platanista*.

Enlarged, crest-like features in the supraorbital and antorbital region are not unique to the platanistoids. Among the odontocetes, several other groups exhibit crest-like features, with the most enlarged forms present in the extant species (perhaps with the exception of some fossil ziphiids) [7,76]. Among these groups are the other so-called river dolphin genera (*Inia* and *Pontoporia*), physeteroids (*Physeter* and *Kogia*), delphinids (*Tursiops*) and ziphiids (*Mesoplodon* and *Hyperoodon*). However, the nomenclature and homology of these facial crests have been confused and debated for centuries. In their lexicon of odontocete skull morphology, Mead & Fordyce [54] discriminate and elucidate these types of crests, ridges, tubercles in the antorbital and supraorbital region; their table 1 is an especially useful rubric for tracing the changing terminology used by previous authors for these features. Our description of the variation in supraorbital crest morphology among the platanistoids expands on these observations made by Mead & Fordyce [54], and provides the first quantitative data describing the variation in this group.

With only one known specimen of *Dilophodelphis*, we cannot be certain of the level of intraspecific variation in crest morphology, but we can comment on the intraspecific variation seen in the crests of other odontocetes. The crests of *Hyperoodon*, which are used in antagonistic ‘head-butting’ behaviours, are larger in males [77] and grow larger and flatter during ontogeny [78]. Dissimilarly, sexual dimorphism in the crest morphology of *Platanista* is minimal [73]. Anderson [79] stated that the crests of both adult and juvenile *Platanista* are ‘more or less quadrangular’ but that slight changes in shape occur over ontogeny that constricts the crests posteriorly in adults. In a physically immature state, *Dilophodelphis* appears to have well-developed crests, but it is possible that these could become even larger in older individuals, or that the morphology may differ between males and females of the species. With the discovery of more fossils of this species, we may gain a better understanding of intraspecific variation in crest morphology, which may in turn shed light on the function of these crests.

### 4.2. Morphological comparisons

Beyond the features associated with the supraorbital crests, *Dilophodelphis* is clearly differentiated from all other platanistids by a number of characteristics. Overall, *Dilophodelphis* is most similar in outward appearance, shape and size to *Araeodelphis*, based on the referred partial cranium collected from the Calvert Formation described by Godfrey *et al*. [19]. Even so, *Dilophodelphis* is clearly differentiated from *Araeodelphis* by the thickness of rostrum—while *Araeodelphis* has a rostrum consistently wider than deep, the rostrum of *Dilophodelphis* is at times equal in width and depth (i.e. at the midlength of the rostrum). *Araeodelphis* also lacks the dorsoposterior sweeping of the alveolar groove as it approaches the rostral base, as seen in *Dilophodelphis*. *Dilophodelphis* further differs from *Araeodelphis* in the shape of its premaxillae posteriorly, which are transversely constricted in *Dilophodelphis*. *Dilophodelphis* also has more pronounced sinistral asymmetry than *Araeodelphis*, with the vertex skewed sinistrally past the midline of the skull.

*Dilophodelphis* differs from all members of the putative group Pomatodelphininae (i.e. *Pomatodelphis*, *Zarhachis* and *Prepomatodelphis*) most prominently in their rostral features. Pomatodelphininae are characterized by extreme longirostry, with a dorsoventrally flattened rostrum and a transversely expanded posterior end of the premaxilla [75]. By contrast, *Dilophodelphis* exhibits a rostrum moderate in length, with no pronounced dorsoventral flattening, and transversely constricted posterior ends of the premaxilla. *Dilophodelphis* also has much greater asymmetry in the vertex than pomatodelphinines.

Finally, beyond their differences in crest shape, *Dilophodelphis* differs from *Platanista* in the following ways: less extreme reduction of the bony orbit, no extreme enlargement/deepening of the posterolateral sulcus, rostrum not transversely flattened, and less reduction of the nasal bones. *Dilophodelphis* also has a wider rostrum base, and a posterodorsal sweep of the alveolar groove as it approaches the rostrum base, both of which are absent in *Platanista*.

### 4.3. Phylogenetics

Our analysis supports the concept of a monophyletic Platanistoidea, which includes the living *Platanista*, and a cluster of allied subclades that consist of three nominal families (Allodelphinidae, Squalodelphinidae and Platanistidae), as well as *Waipatia*, *Otekaikea* and possibly *Squalodon*. Platanistoidea (excluding *Squalodon*) is supported by six synapomorphies: emargination of the posterior edge of zygomatic process by neck muscle fossae, skull in lateral view [character 52(1,2)]; the presence of suprameatal pit of squamosal [character 57(1,2)]; the presence of posterior periotic fossa of squamosal [character 59(1,3)]; lateral groove or depression affecting profile of periotic as viewed dorsally [character 60(1)]; anteroposterior ridge on dorsal side [character 61(1)]; ventral surface of posterior process of periotic concave or convex along straight path perpendicular to its long axis [character 66(1,2)].

By mapping both the presence or absence of crest pneumatization and the relative size of the crests onto our strict consensus phylogenetic tree, we were able to visualize these traits in an evolutionary context (figure 7). Our analysis shows that both enlarged crest size and pneumatization evolved only once, and in one clade (Squalodelphinidae + Platanistidae), but at different times. The enlargement of the supraorbital crests began in the Late Oligocene [74], with the Squalodelphinidae evolving modest-sized crests. In the Early Miocene, supraorbital crests reached extreme sizes among the platanistids. However, the platanistids evolved enlarged crests without the invasion of air sacs, as seen in *Dilophodelphis*. Pneumatization first appears in the pomatodelphinines in the Early Miocene, with the appearance of *Zarhachis.* Therefore, the evolution of pneumatization is de-coupled from the enlargement of the supraorbital crests among the platanistoids.

### 4.4. Functional morphology

The morphology of *Dilophodelphis* raises several questions about the function of supraorbital crests in platanistoids. Our only extant model for the function of supraorbital crests in platanistoids is the South Asian river dolphin (*Platanista gangetica*), whose prominent, concave crests are hypothesized to serve a role in echolocation because they support maxillary sinuses that lie dorsal to the melon, though experimental determination remains hard to achieve. In other odontocetes, sound produced by the phonic lips is reflected or absorbed by either the air sac system surrounding the nasal passages, or the crests themselves, producing a more anteriorly projected acoustic signal [80,81]. For *Platanista*, Pilleri [82] referred to this system as a ‘sound screen’ that prevents sound from passing dorsolaterally from the caudal half of the melon. *A priori* we would expect that crests used to reflect sound anteriorly would have a highly concave medial surface (allowing them to focus reflected sound rather than dissipating it) and that they would be denser (which would allow for greater acoustic impedance mismatch between soft tissue and bone). The density and morphology of the crests in *Platanista* certainly meet these requirements. However, the internal surface of the crests of *Platanista* are highly convoluted, which would prevent reflection of sound and instead may allow for sound to be better absorbed by the bone. Because of this configuration, we infer that if *Platanista* reflects sound anteriorly using its crests, it is probably doing so using the sinuses lining the crests rather than the bone itself. The acoustic mismatch between soft tissue and air is much lower than that between air and compact bone though, which logically would be a less effective method of sound reflection. More basic anatomical work on this highly endangered taxon is necessary to refine and ground truth these hypotheses.

**Figure 10.**
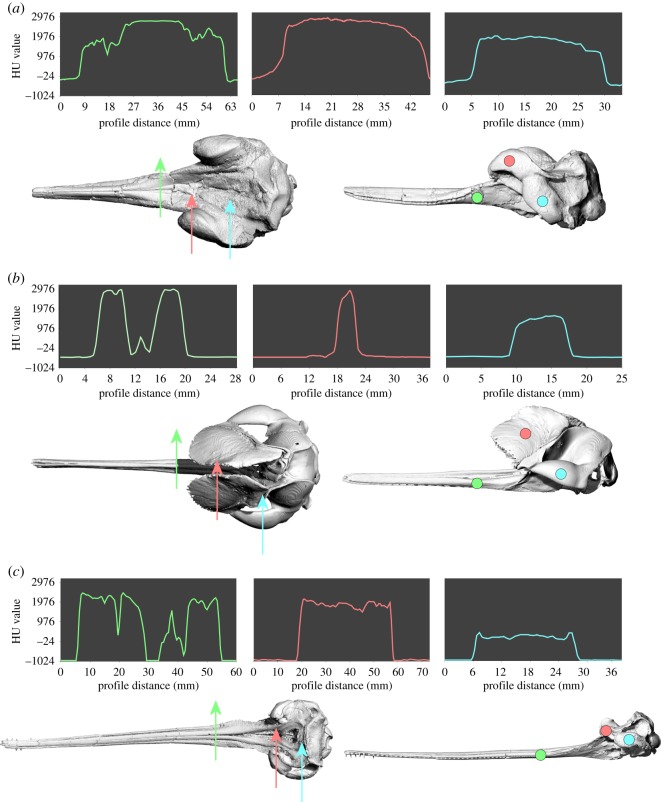
Relative density (Hounsfield units). Measures were taken as lateral profiles through the rostrum (green), crests (red) and zygomatic process (blue). (*a*) *Dilophodelphis* (USNM 214911), (*b*) *Platanista gangetica* (USNM 23456) and (*c*) *Pomatodelphis inaequalis* (USNM 187414). Dorsal and lateral views of the skulls show the position where density profile lines were taken.

The morphology of *Dilophodelphis*' crests differ substantially from those of *Platanista*. They are robust and both laterally and dorsally expanded. Their medial surface is convex, a characteristic that would make it difficult for these structures to focus sound in any certain direction. The density of the crests of *Dilophodelphis* is similar to that in other platanistoids. In all species included in our analysis, the crests were denser than the zygomatic process but of similar density to the maxillae within the rostrum. The crests of *Dilophodelphis* are slightly denser than those of *Platanista* and *Pomatodelphis* compared with the other regions of the skull. Unfortunately, this finding may not tell us much about possible variation in crest function in these taxa (figure 10). Diagenetic processes may also influence these results and so care must be taken when drawing conclusions from small differences in density. Also, it may be that most of the acoustic impedance is undertaken by the maxillary sinuses rather than the bone itself, and hence bone density may not be an important factor in determining the performance of the system.

There are multiple possible functions for the large crests of *Dilophodelphis*. First, these crests could support soft tissue structures or sinus systems in a similar way to *Platanista*, but the relative size of the crests seems overdesigned for this purpose. Without a sinus system to absorb or reflect sound, it is possible that bone needed to be thicker to adequately perform the same function as a sound screen. Second, crests of this size could act as protection, providing structural reinforcement for loads that occur during feeding, evading predators or interspecific fighting (i.e. competition). However, it is hard to imagine a scenario where loads are applied to this region of the cranium. The long, slender rostrum would be more likely to engage with an object rather than the dorsal surface of the head. Third, the crests may act as display structures. Many species of odontocetes such as male beaked whales possess elaborate display structures that allow for intraspecific or interspecific signalling. It has even been hypothesized that odontocete species may be able to sense differences in internal morphology using echolocation [83]. However, this hypothesis has mainly been applied to ziphiids, where the facial and rostral bones are significantly more compact than in *Dilophodelphis*. The greatly enlarged surface area of the maxilla in the supraorbital region is known to serve as an attachment point for the highly modified maxillonasolabial muscles, which are involved in sound production [5]. Theoretically, crests could also act as greater attachment locations for these facial muscles, including those that attach to the melon, blowhole and other soft tissue structures of the forehead. While the robust crests of *Dilophodelphis* seem overdesigned for this purpose, the height of the structures could provide orientations for muscle fibres necessary to control the shape of the forehead elements [6]. To the best of our knowledge, there has been no study of how the elaboration of the crests in *Platanista* contributes to facial and rostral muscle attachments. Lastly, the crests may provide space for sinuses. Mammals have evolved, or through ontogeny, developed sinus systems for a wide range of functions including weight reduction, space for chamber resonance or thermoregulation [84]. Here we find no compelling evidence of a sinus system on the medial surface of the bone in *Dilophodelphis*, but think that this hypothesis is worthy of consideration given the presence and importance of the sinus system in *Platanista.* Future studies could assess these hypotheses using a variety of techniques. Modelling approaches, such as finite-element analysis, offer a useful mechanism to test the structural, acoustic and thermoregulatory benefits of different crest morphologies. Further work on the function of soft tissues in the acoustic system of *Platanista* would also greatly benefit any interpretation of crest function in fossil taxa.

## Institutional abbreviations

LACM: Departments of Mammalogy and Vertebrate Paleontology, Natural History Museum of Los Angeles County, Los Angeles, CA, USA.

USNM: Departments of Paleobiology and Vertebrate Zoology (Division of Mammals), National Museum of Natural History, Smithsonian Institution, Washington, DC, USA.

YPM: Division of Vertebrate Paleontology, Yale Peabody Museum, New Haven, CT, USA.

## Supplementary Material

Reflectivity of convex vs. concave surfaces

## Supplementary Material

Dataset of platanistoid crest measurements

## Data accessibility

The 3D file of the holotype USNM 214911, along with the original DICOM files, is archived at Zenodo (http://zenodo.org) at the following doi: 10.5281/zenodo.232492. The 3D model of *Dilophodelphis* is also available for viewing and download on the Smithsonian X 3D website (http://3d.si.edu).
