# Supplementary figures and images for: A new fossil dolphin Dilophodelphis fordycei provides insight into the evolution of supraorbital crests in Platanistoidea (Mammalia, Cetacea)
Source: R Soc Open Sci. 2017 May 31;4(5):170022. doi: 10.1098/rsos.170022 (PMC5451807; doi:10.1098/rsos.170022)

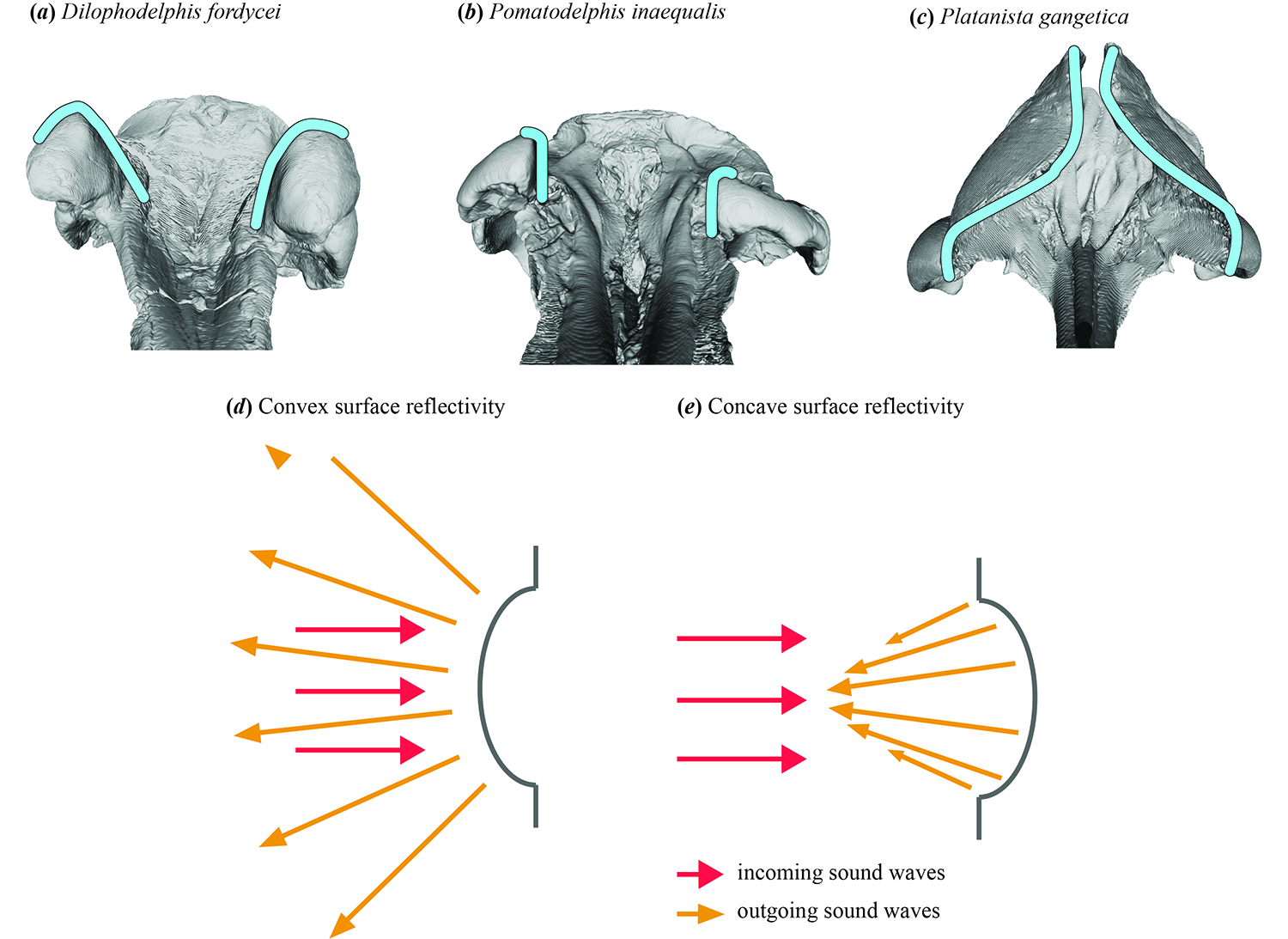

Supplement: Reflectivity of convex vs. concave surfaces [file rsos170022supp1.tif]
